# Quantifying anterior pelvic roll during total hip arthroplasty in the lateral decubitus position

**DOI:** 10.1186/s13018-023-04350-y

**Published:** 2023-11-14

**Authors:** Andrew P. Kurmis, Ernest C. Lourens

**Affiliations:** 1 Discipline of Medical Specialties, University of Adelaide, Adelaide, SA Australia; 2 Department of Orthopaedic Surgery, Lyell McEwin Hospital, Haydown Road, Elizabeth Vale, SA 5112 Australia; 3 College of Medicine and Public Health, Flinders University, Bedford Park, SA Australia; 4 Department of Orthopaedic Surgery, Mount Gambier Hospital, Mount Gambier, SA Australia

## Abstract

**Background:**

Unintended pelvic positional change is an acknowledged intra-operative problem for hip arthroplasty, seen commonly with procedures performed in the lateral position. If unrecognised, such changes can dramatically alter final acetabular component anteversion potentially resulting in suboptimal construct performance. It has previously been suggested that pelvic roll of just 13° may be enough to place an otherwise perfectly orientated cup outside of conventional ± 10° safe zones. Using the real-time tracking capacity of a commercially available optical navigation system, we aimed to accurately quantify pelvic roll occurring during total hip arthroplasties (THAs) performed in the decubitus position.

**Methods:**

Prospectively collected data for 107 consecutive, unilateral, THAs were interrogated to determine the magnitude of pelvic movement around a central longitudinal axis (i.e. AP roll). Correlation statistics with patient age and body mass index (BMI) were also calculated.

**Results:**

A mean pelvic roll of 9.5° was observed, being anterior in 96% of cases. Of these, 18.3% of hips had a magnitude of roll greater than 13°. There were no statistically significant independent correlations observed between age (*p* = 0.87) or BMI (*p* = 0.59) and mean roll.

**Conclusions:**

Errors in achieving acetabular target version may result in numerous post-operative concerns including instability/dislocation, bearing wear, squeaking, range-of-movement limitation and increased revision rate. In a general cohort, our findings suggest a mean anterior pelvic roll during THA of nearly 10°. Without purposeful correction, this may cause substantial deviation from intended target positions. Future work is indicated to map changing pelvic roll during THA which is likely to follow a nonlinear trajectory.

*Level of evidence***: **IV.

.

## Introduction

Achieving optimised final insertion position of the acetabular component of primary total hip arthroplasties (THAs) remains a critical determinant of many tangible outcomes associated with function and implant survivorship [11, 12]. While the ‘ideal’ definitive position for an individual patient remains contentious, most surgeons agree that the ability to consistently achieve attainment of the desired ‘target’ orientation (whatever that may be) is an important procedural skill. One recognised barrier to placing the component in the intended target position is unanticipated or unappreciated change in pelvic position throughout the operation up until the point of definitive cup placement [7]. Such intra-operative movement occurs more commonly in the lateral (decubitus) set-up. A recent structured review of the literature suggested that such positional movement during THA surgery is common and may be routinely of great enough magnitude to result in final cup position outside of accepted ‘safe zones’ [7]. The aim of this study, using a highly precise optical navigation system, was to quantify the amount of pelvic roll seen during routine THAs performed in the lateral decubitus position.

## Methods

Prospectively collected data from the local database of a single surgeon series’ of computer-navigated THAs were retrospectively interrogated. All registry data were patient de-identified at the time of index entry, thus no patient-identifying information were utilised or accessed for this study. A convenience sample of 107 consecutive computer-navigated THAs were extracted from the registry which included adult patients (i.e. age at the time of surgery ≥ 18 years) undergoing unilateral procedures performed in the lateral position at one of two metropolitan tertiary teaching hospitals. Patients undergoing excisional arthroplasty, first-stage revision, or simultaneous bilateral THAs were excluded. Local human research ethics committee requirements were met in the performance of this study.

The magnitude of pelvic roll—as previously defined as the angular change in position around a central longitudinal axis [7]—was extracted manually for each patient from the available dataset. Collected data were tabulated separately into a Microsoft Excel (Microsoft Corporation; Redmond, WA, USA) spreadsheet and analysed using the SPSS (IBM; Armonk, NY, USA) statistical software. Statistical significance was set a priori at 0.05. Pearson correlation coefficient testing was performed to determine the association between the mean anterior pelvic roll and both age and BMI, independently.

## Surgical technique

**Fig. 1:**
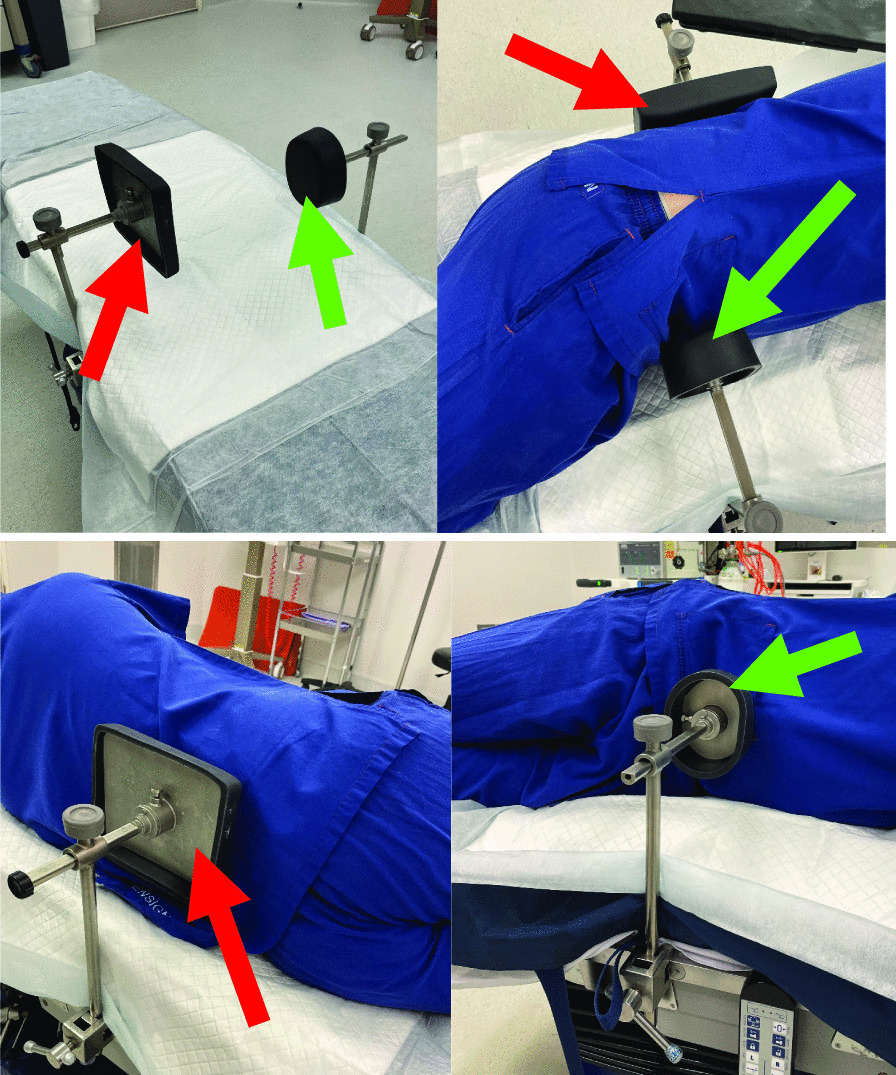
Positioning support set-up demonstrating rectangular posterior (sacral) [red arrow] and rounded anterior (symphyseal) [green arrow] bolsters

After appropriate anaesthetic induction, all THAs were performed in a lateral decubitus position. All operations (100%) were performed using routine positioning clamps which included a generic curved (rectangular) adjustable bolster overlying the sacrum posteriorly and a single-posted round anterior bolster positioned against the symphysis pubis (Fig. 1).

**Fig. 2:**
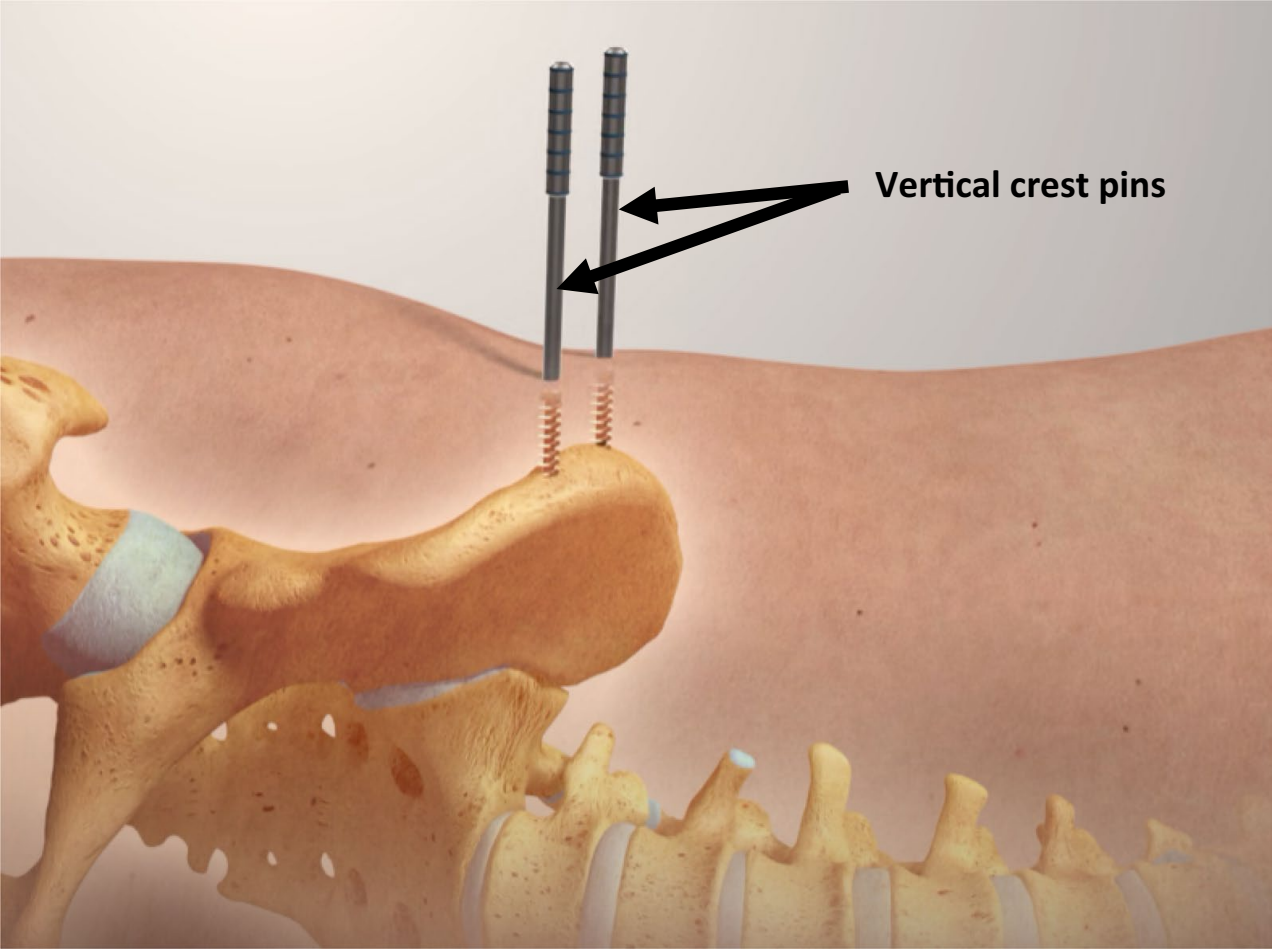
Insertion of two 4.5-mm self-drilling, self-tapping, threaded pins into the ipsilateral iliac crest. Starting point 2–3 cm posterior to the anterior superior iliac spine (ASIS)

**Fig. 3:**
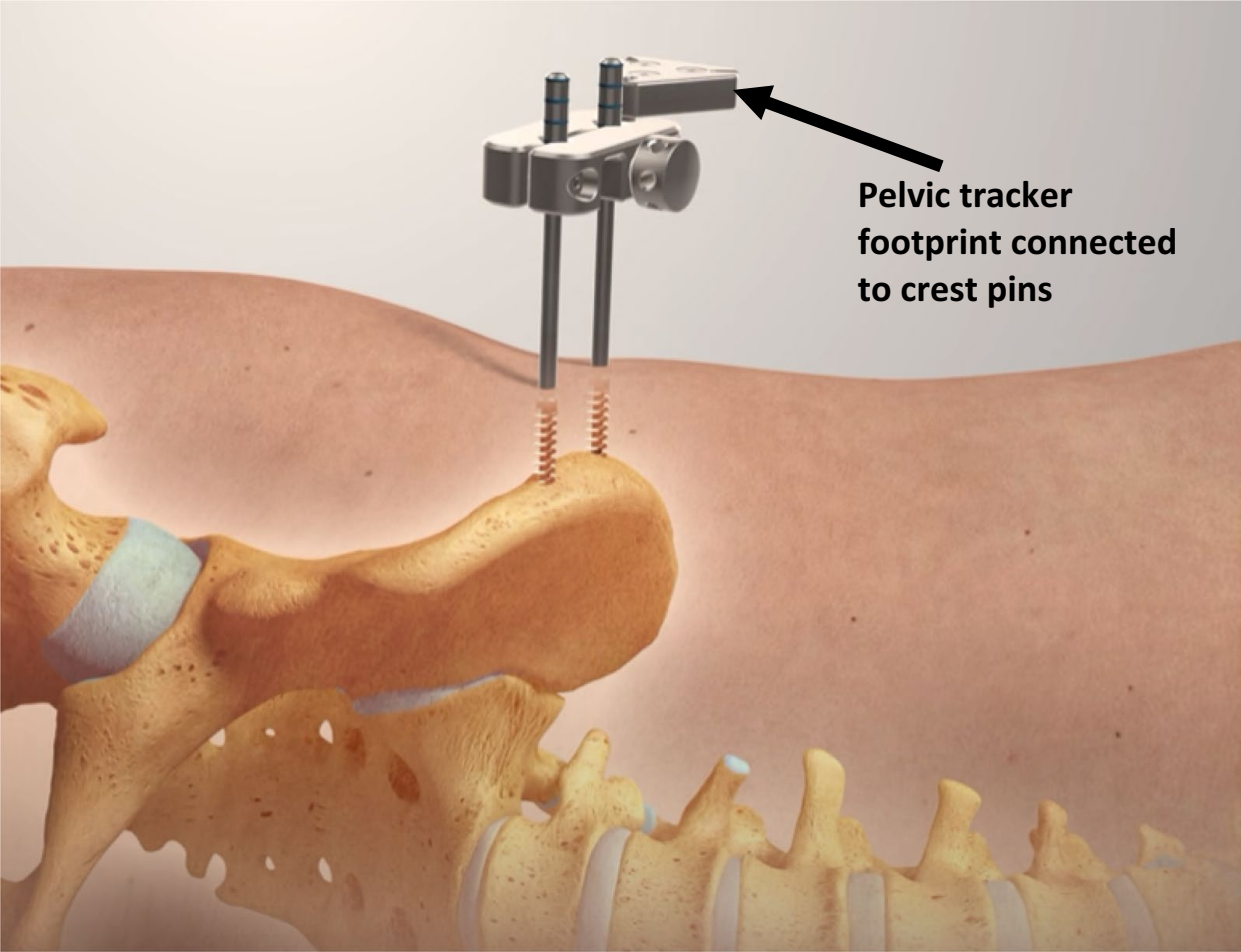
Engagement of the pelvic tracker magnetic footprint to the in situ crest pins

**Fig. 4:**
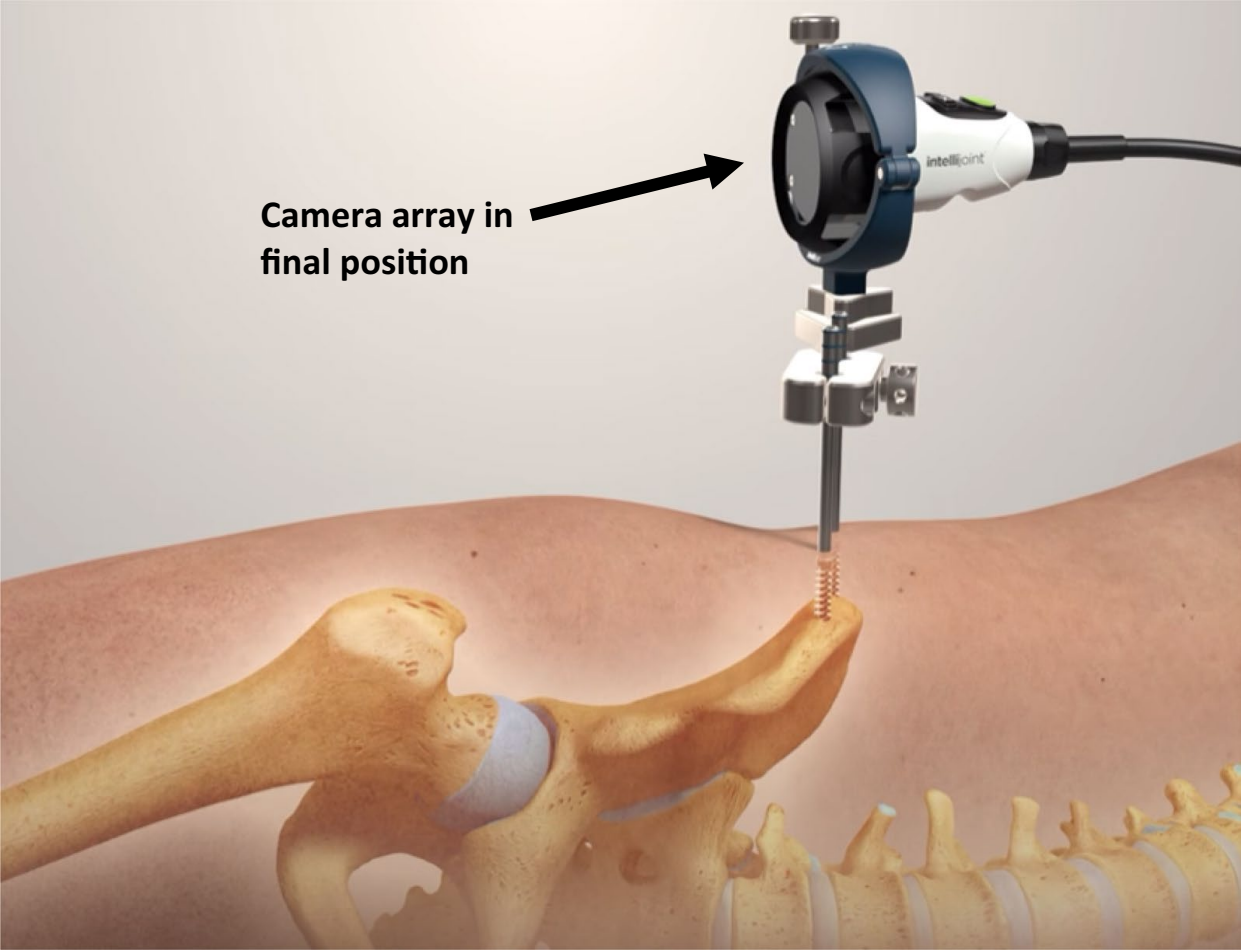
Intellijoint HIP® camera array in position on pelvic tracker platform

The ‘true’ lateral starting position was determined using a composite of the verticality of the anterior superior iliac spines (ASISs) and sacrum, and a horizontal central gluteal cleft/fold. After pre-scrub, standard skin prepping and sterile draping as per the local convention, the procedure commenced with insertion of a fixed pelvic tracker platform (Intellijoint; Intellijoint Surgical, Ontario, Canada) into the anterior element of the ipsilateral iliac crest. Following the proprietary surgical technique, this tracker consists of two self-drilling and self-tapping 4.5-mm threaded pins which were introduced perpendicular to the lateral table of the iliac crest and advanced to achieve bicortical fixation [8] (Fig. 2). The first (anterior) of these two pins was inserted under power through a tissue-protecting sleeve approximately 2.5 cm behind the ASIS into the solid bone stock behind the traction epiphysis marking the origin of the sartorius muscle. The second (posterior) crest pin was inserted approximately 2 cm posterior to the first, along the broadest element of the iliac crest in this region. The basic perpendicular stability of each pin was confirmed before the two pins are linked by the fixed tracker footprint device which was manually attached to the two pins and tightened by hand (Fig. 3). At this point, the system camera was applied to the tracker base (Fig. 4) and was calibrated to the starting position. The system incorporates a high-precision, integrated digital accelerometer, which is part of the Intellijoint fixed camera assembly—determinations of pelvic movement (i.e. roll) are made directly from this unit, independent of any in-field trackers. The operative leg was returned to a ‘neutral’ position with the heels and knees superimposed to the underlying contralateral limb. This measure of orientation in three-dimensional (3D) space served as the ‘zero’ starting point and permitted later comparison for movement of the pelvis. It is noteworthy that attainment of a ‘true’ lateral decubitus position has no direct bearing on subsequent measurement, rather the difference between the initial and later positions was used to reflect unintended pelvic ‘movement’. The operation was then performed as per the clinical workflow of the operating surgeon. While the system continuously monitors pelvic roll throughout the case (and this can be displayed in real time if so desired), data capture for final record keeping purposes requires manual activation of a ‘capture’ button either via the camera assembly unit (surgeon control) or system laptop (outside of the sterile field).

The ‘final’ measurement of pelvic roll was performed immediately after all definitive implant components had been inserted, the prosthetic joint safely reduced, and the operative leg returned to a neutral position.

## Results

The results for 104 of the 107 cases were available for review with ‘final’ pelvic position measurements not available in three cases. Excluded cases included two situations where the pelvic tracker pins were deemed to have become mechanically loose during the case with the resultant measurements deemed grossly inaccurate. In one other case, the navigation software shut down mid-procedure secondary to a broken power cable to the laptop running it, resulting in unrecoverable data loss. Intention-to-treat data inclusion principles were employed for analysis.

The mean age of patients at the time of surgery was 71.3 years (range 47.4–93.9), with 54.8% female (57/104). Accurate BMI data were only available in 101 of 104 cases. Of those patients with accurate height and weight data recorded, the mean BMI was 29.4 (range 16.6–43.0). Six (6) patients had BMIs of 40 or greater. Forty-three (43) patients underwent a general anaesthetic (GA) with 61 undergoing instead spinal anaesthesia and titrated sedation. The primary indication for surgery was osteoarthritis (OA) in 62.7% of cases, avascular necrosis (AVN) in 22.6%, neck of femur fracture in 10.7%, and ‘other’ in 4.8%.

**Fig. 5:**
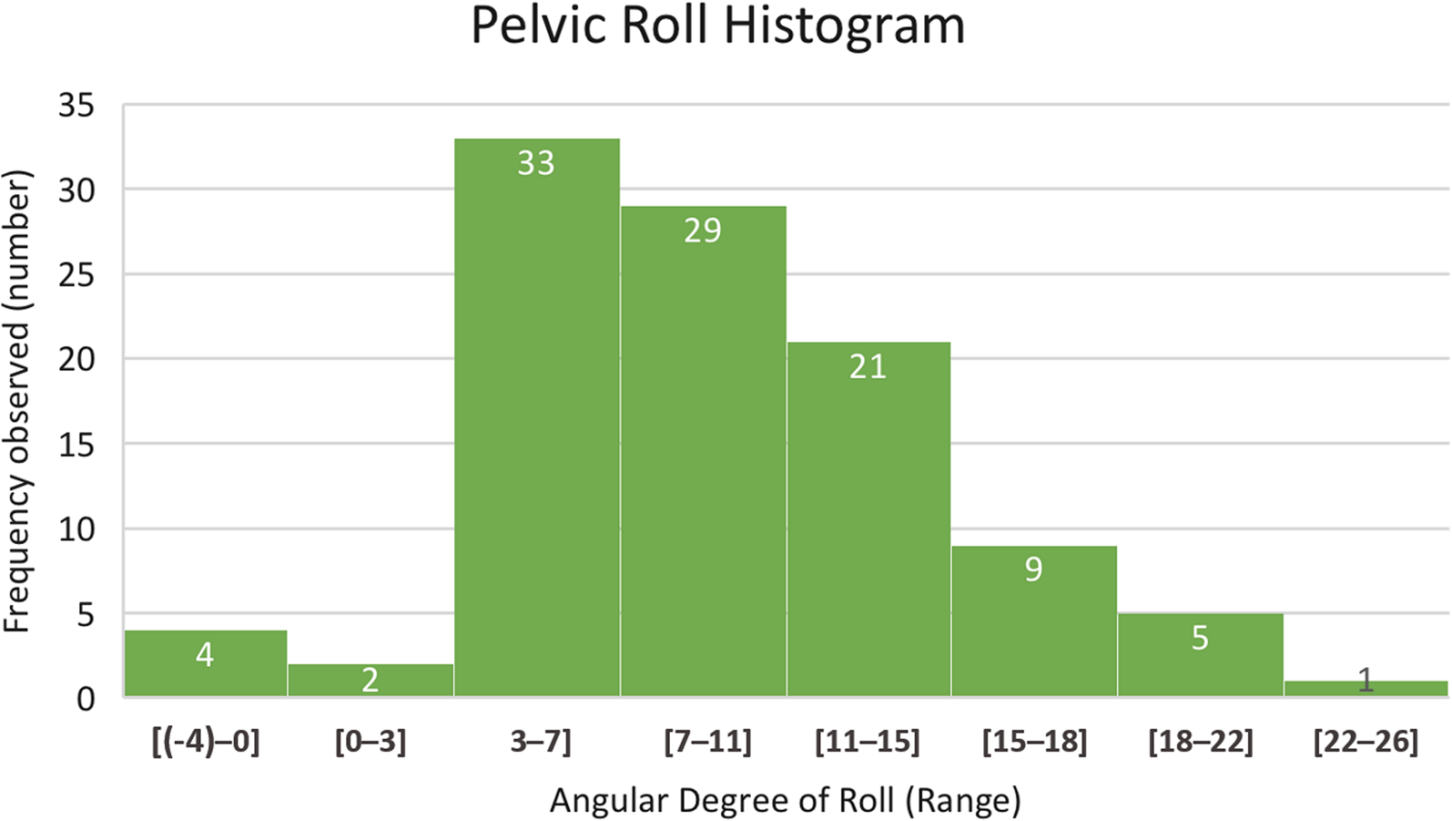
Cohort distribution of pelvic roll (including vector), showing median anterior roll of 3–7°

Across the entire cohort, the mean pelvic roll was 9.50° (range 1.0–25.0°). The distribution of pelvic roll across the cohort is shown in Fig. 5. In the majority of cases (96%), this roll was anterior in vector, with a median anterior roll of between 3 and 7°. There were no statistically significant independent correlations observed between age (*R* = 0.0159; *p* = 0.8727) or BMI (*R* =  − 0.1889; *p* = 0.5851) with regard to the mean magnitude of unintended movement around the central longitudinal axis.

## Discussion

The majority of primary THAs worldwide continue to be performed in the lateral (decubitus) position [1]. While there remains surgeon variation in the landmarks and techniques utilised to achieve final intra-operative position of the acetabular component of a THA—and also in the ‘ideal’ target position for an individual patient—it is widely accepted that the ability to place the cup consistently as close as possible to the desired target position is important for post-operative outcomes [7].

Previous research has suggested that unintended pelvic positional change does occur during routine THA surgery and that surgeons and surgical teams are poor at recognising this with any degree of consistency [7]. It has also been suggested that contemporary positioning devices to support THAs in the lateral position may unreliable [4, 5] and perform poorly with regard to consistent maintenance of the neutral (i.e. starting) position [7, 13]. Additionally, such devices are also associated with a not inconsequential morbidity profile, including medically important local skin pressure ulceration [6, 16].

Utilising an off-the-shelf commercial THA computer-navigation system, we have been recording for over 2 years now pelvic roll data for patients undergoing hip arthroplasty. The precision of angular and directional measurement accuracy of this system has previously been validated by others and reported to be less than 1° and 1 mm, respectively [15]. Given our recently published structured review of the topic of intra-operative anterior pelvic roll [7], we undertook the current investigation to provide contemporary, quantitative data regarding such unintended movement during routine surgery. This validated and widely used imageless optical system allowed us to recognise and quantify pelvic movement with a high degree of measurement precision [15].

Limited previous studies have quantitatively explored unintended movement around a central longitudinal axis during THA in the conventional lateral position. Our key finding of a mean anterior pelvic positional roll during THAs of 9.50° does however compare favourably to others who have suggested similar movements. The earlier 2014 work by Grammatopoulos et al. [4] prospectively recruited 67 patients who underwent high-precision intra-operative stereophotogrammetric (SPG) measurement analysis during routine primary THA, allowing static assessment of pelvic positional change from initial set-up to post-cup implantation. Of the 50 patients who underwent a conventional posterior approach, a mean pelvic roll of 9° was observed. There is no evidence contrasting the true precision of SPG (which relies on orthogonal projection measurement) versus modern optic navigation systems, although the close similarity of our respective findings—collected nearly a decade apart—suggest the problem of unintended pelvic movement is not a new phenomenon.

The more recent work by Della Valle and colleagues (2019) [2] reviewed changes in intra-operative pelvic position during primary THA of 75 patients, operated on by four different surgeons, also using imageless, intra-operative navigation. All patients were operated on using a ‘devoted’ surgical operating table and standardised single contact area posterior (i.e. sacral) and anterior (i.e. pubic) compression posts. This largely mirrors the technique we have employed in our own study (and that which we use in non-research conditions also). These authors reported an average maximum anterior pelvic roll of 17.62° which peaked immediately before final cup insertion. They also highlighted that pelvic roll appeared to partially ‘correct’ progressively towards the neutral starting position thereafter during the operation—likely due to the imparted forces of cup impaction and joint relocation. They suggested therefore that the ‘final’ measured pelvic movement during a case performed in the lateral position may not accurately reflect the maximal angular change which had otherwise occurred.

Previous authors have proposed acceptable ‘cut offs’ to define clinically important variation [13] in intra-operative pelvic roll. Anterior (or posterior) pelvic tilt alters the position of the cup in the sagittal plane [17] which has a direct impact on surgical version perception. In case series’ including 67–100 hips [2, 4, 5, 13, 16], previous works have reported 41–57% of cases rolling anteriorly > 5 degrees [5, 9, 13], with 21–38% by > 10 degrees [3, 13, 14]. Otero’s 2018 paper reported 15.4% of cases with 10–20° of anterior roll and 2.8% with > 20 degrees [14]. It is noteworthy that these studies largely reported end of case change (versus the starting position) which—as was highlighted in Della Valle et al.’s work (2019) [2]—unlikely represents the true ‘maximal’ pelvic movement occurring.

Langston and colleagues in 2018 [10] suggested that an uncorrected/uncompensated change in pelvic position of 13° or more may be considered unacceptable as this will result in a change in the functional anteversion of the acetabulum of 10°. As a result, there is a possibility that even a supposedly well-aligned component will be placed outside of a safe target zone of ± 10° which may have consequences for later construct instability and dislocation risk. Comparing these reports to our own findings, we observed 18.3% (19/104) of our hips with a magnitude of roll greater than 13°.

In our moderately sized prospective cohort, we were unable to demonstrate a statistical relationship between patient BMI and the magnitude of pelvic roll (p = 0.5851), although recognising that our investigation was not powered specifically to do so. It may be possible that a true relationship between these two variables does exist and that simply our study was not large enough to demonstrate this. Dedicated studies exploring the impact of increasing BMI of unintended pelvic movement therefore represents an opportunity for future work.

We acknowledge several limitations to our study. Firstly, the retrospective nature of our work. While we would contend that all data utilised for the analyses contained herein were prospectively collected in the index setting, the possibility for bias due to ad hoc use is recognised. Secondly, although the data we have utilised were drawn from the local database of a single consultant arthroplasty surgeon, the final ‘positioning’ determination of the patient in each case was often made by registrar/fellow-level clinicians at various stages of training experience. There may be an influence on the degree of movement reflective of the clinical experience level of the individual performing the final (pre-draping) positional and support device checks—our database did not allow retrospective determination of this. Thirdly, all cases were performed using a standardised rectangular sacral (posterior) and round anterior (symphyseal) bolster. Although previous work [3, 4] has suggested great inconsistency in most conventional positioning devices, we have no way of confirming the wider generalisability of the devices used locally. Comparative studies using a variety of available such devices will shed light on this. Finally, while our work does provide robust evidence of the mean extent of anterior pelvic roll at the end of a hip arthroplasty case, it does not provide discriminatory information as to at which specific stages during the workflow of the procedure this movement occurred and whether such movement is linear in nature across the case. This again represents an avenue for future, more detailed, investigation.

## Conclusions

Unintended change in the position of the fixed pelvis during THA in the lateral position has the potential to introduce perceptual error in final acetabular component placement. Biomechanically, this may result in several post-operative concerns including instability/dislocation, bearing wear, squeaking, range-of movement limitation and increased revision rate. Using the real-time tracking capacity of a commercially available, highly precise, imageless, intra-operative optical navigation system, our findings suggest a mean anterior pelvic roll during routine THA of nearly 10°. Such unanticipated movement directly alters the effective anteversion of cup placement and—without purposeful correction—may result in substantial deviation from the intended target position. Future work is indicated to better map the change in pelvic roll during THA which is likely to follow a nonlinear trajectory.

## Data Availability

Not applicable.

## Abbreviations

3D: Three dimensional
AP: Anteroposterior
ASIS: Anterior superior iliac spine
AVN: Avascular necrosis
BMI: Body mass index
GA: General anaesthetic
OA: Osteoarthritis
SPG: Stereophotogrammetric
THA: Total hip arthroplasty
